# Supplementary material for: Drosophila Embryonic Hemocytes Produce Laminins to Strengthen Migratory Response
Source: Cell Rep. 2017 Nov 7;21(6):1461–70. doi: 10.1016/j.celrep.2017.10.047 (PMC5695906; doi:10.1016/j.celrep.2017.10.047)
Supplement: Document S1. Figures S1–S5 [file mmc1.pdf]

Cell Reports, Volume *21*

Supplemental Information

Drosophila Embryonic Hemocytes Produce Laminins to Strengthen Migratory Response

Besaiz J. Sánchez-Sánchez, José M. Urbano, Kate Comber, Anca Dragu, Will Wood, Brian Stramer, and María D. Martín-Bermudo

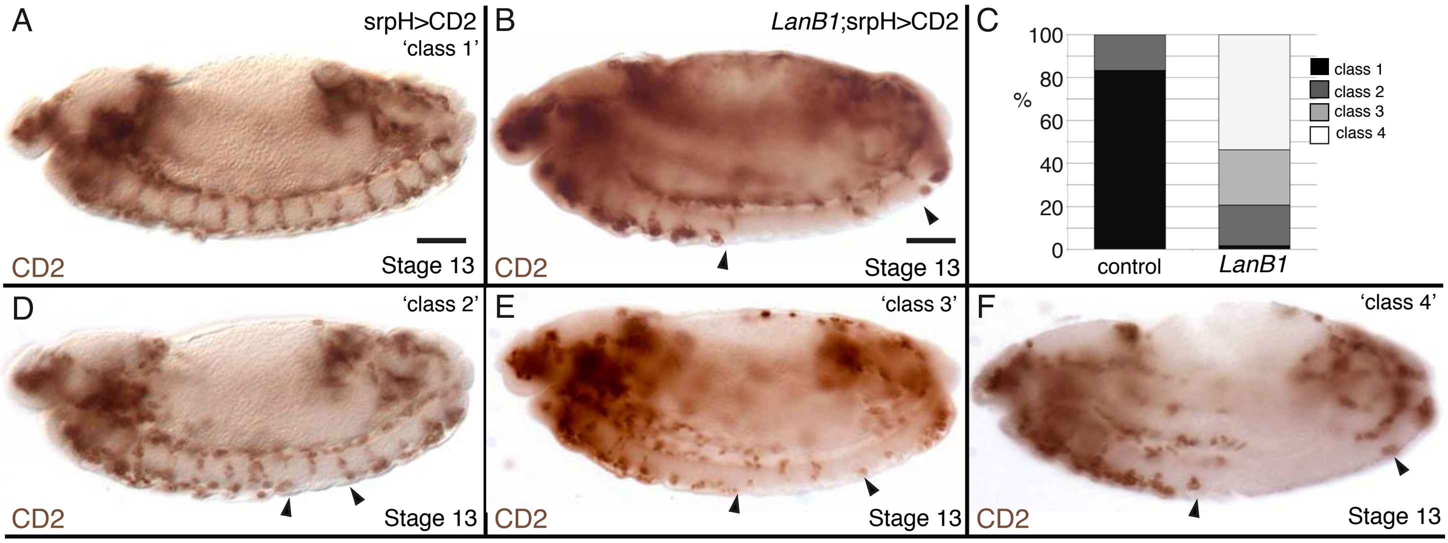

Supplemental Figure 1 (related to Figure 1): Hemocyte migration is defective in the absence of laminins.

(A, B, D-F) Lateral view of fixed and stained stage 13 embryos. Hemocytes are visualized by expression of the heterologous cell membrane marker CD2 driven by the srpH-Gal4 driver and detected with an anti-CD2 antibody. (A) Control embryo. (B) LanB1 embryo. (C) Quantification of hemocyte migration phenotype over the VNC. This was done accordingly to (Comber et al., 2013). To summarize, we used four distinct phenotypic classes, according to the number of neuromites of the VNC devoid of hemocytes: 'class 1': 0 neuromites, 'class 2': 1-2 neuromites, 'class 3': 3-4 neuromites and 'class 4': ≥ 5 neuromites. (D-F) Grading of LanB1 embryos into 'classes' based on the migration defects along the VNC.

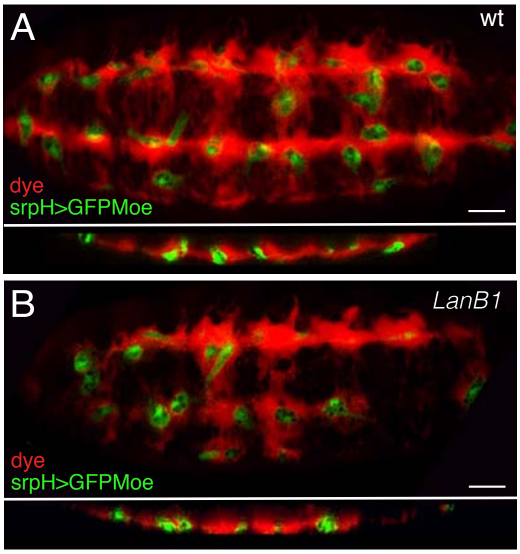

Supplemental Figure 2 (related to Figuress 1 and 2): Hemocyte migration along the VNC: environmental requirements for laminins.

(A, B) Orthogonal projections of ventrally orientated stage 15 control and LanB1 embryos expressing GFP in hemocytes (green) injected with dextran dye (red) to reveal spatial constraints surrounding the hemocytes. While in control embryos (A), the dye permeates along the length of the embryo, indicating proper VNC-epithelial separation, in LanB1 embryos (B), spreading of the dye becomes restricted, demonstrating incomplete VNC-epithelial separation. Scale bar represents 50µm.

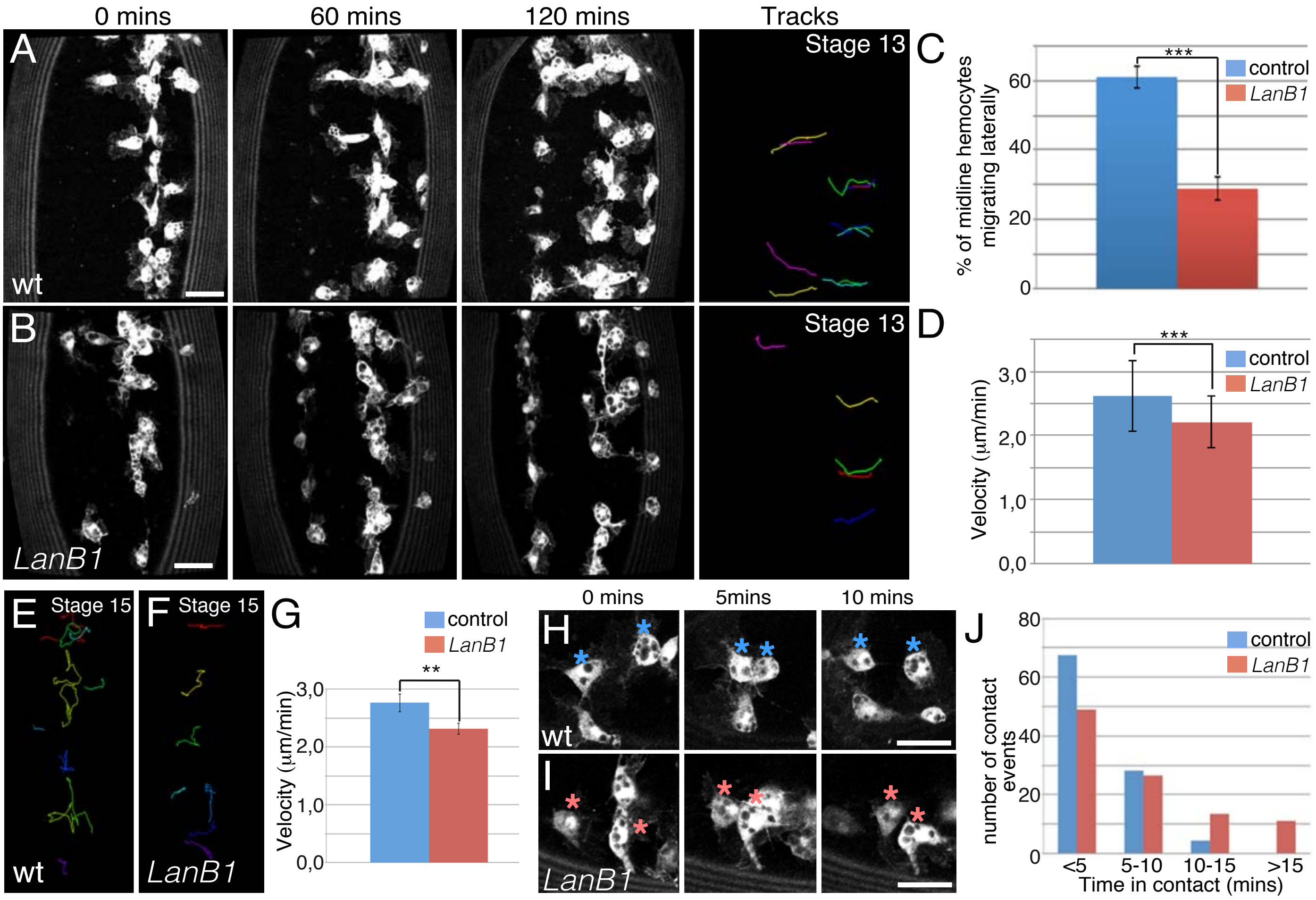

Supplemental Figure 3 (related to Figures 1 and 2): Laminins regulate hemocyte lateral and random migrations

(A and B) Still images taken from live imaging of hemocytes expressing GFPMoe undergoing lateral migration at stage 13/14. (C) Tracking hemocytes undergoing lateral migration reveals a decrease in the percentage of hemocytes migrating laterally in LanB1 embryos (average decrease from 62.2% and 29.4% $p<0.001$, $n=5$ embryos per genotype) and (D) a significant decrease in the speed of the hemocytes that did (average velocity of hemocytes from control and LanB1 embryos were 2.62 ± 0.56 µm/min and 2.21 ± 0.40 µm/min respectively, $p<0.001$). (E, F) Tracking of GFP expressing hemocytes undergoing random migration at stage 15 reveals a slight but statistically significant reduction in random migration velocity of hemocytes from LanB1 embryos compared to controls (G). Hemocytes from control and LanB1 embryos migrated at 2.76 ± 0.15µm/min and 2.31 ± 0.09µm/min respectively, $p<0.01$). (H, I) Stills taken from live imaging of GFP expressing hemocytes in control and LanB1 mbryos undergoing cell-cell contact repulsion at stage 15. Hemocytes remain in contact for longer periods in LanB1 embryos (I, pink asterisks) than in control embryos (H, blue asterisks). (J) Quantification of the time the lamellipodia of two hemocytes remain in contact indicates an increase in LanB1 embryos (average time in contact for hemocytes from control and LanB1 embryos was 4.9 and 7.6 mins respectively, $p<0.001$, $n=91$ (control) and $n=100$ (LanB1) contact events). Scale bar represents 20 µm.

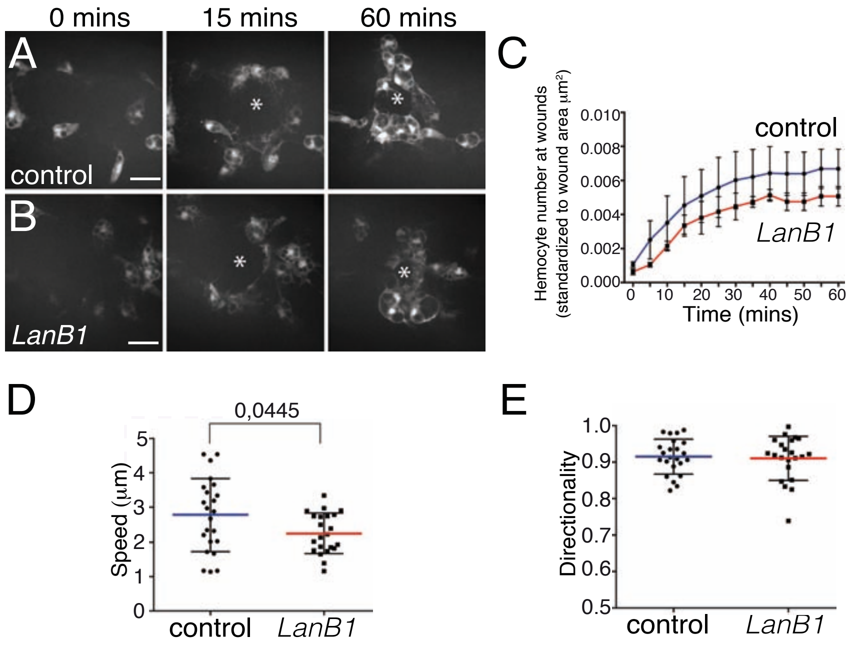

Supplemental Figure 4 (related to Figure 1): Laminins are required for inflammatory migration

(A, B) Stills taken from movies of hemocytes in control (A) and LanB1 (B) embryos migrating to an epithelial wound (asterisk). Scale bar represents. (C) Monitoring the number of hemocytes at the wound every 5 minutes post wounding over a 60 minute time period reveals a small but significant reduction in the number of hemocytes present at early time points following wounding in LanB1 embryos compared to control ($p<0.05$ at 10 and 15 mins post wounding). (D) In addition, tracking reveals that hemocytes from LanB1 embryos migrate towards a wound at lower speed than those from controls (2.3 ± 0.6µm/min and 3.0 ± 0.3µm/min respectively, $p<0.05$, n=23 (control) and n=22 (LanB1) hemocytes). Scale bar represents 25µm.

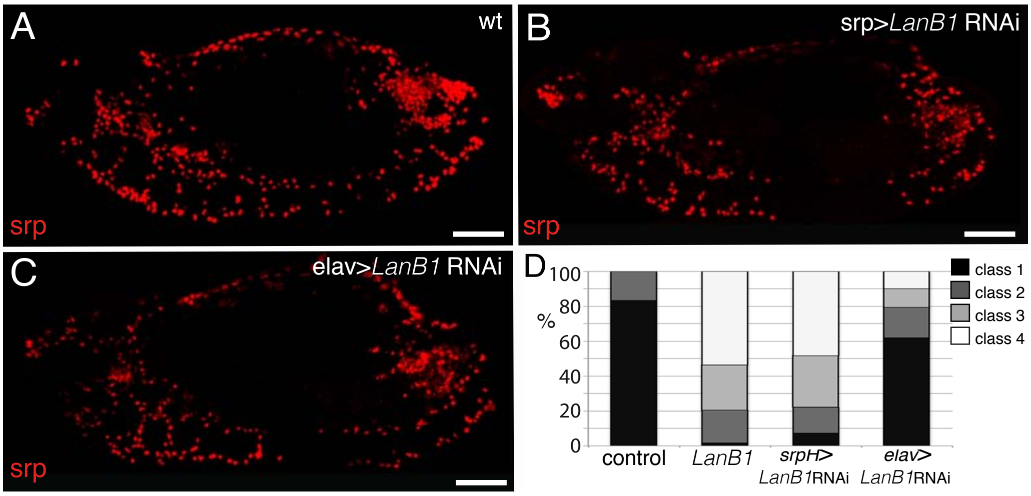

Supplemental Figure 5 (related to Figures 1 and 2): Hemocyte and neuronal specific requirements for laminins

(A-C) Lateral view of fixed stage 13 embryos stained with an anti-Srp antibody (red). (A) Wild type embryo and LanB1 RNAi specific expression in either hemocytes (B) or the VNC (C). Down-regulation of LanB1 levels specifically in hemocytes (B), but not in neuronal and glial cells (C), results in migration defects similar to those observed in LanB1 embryos. (D) Quantification of the hemocyte migration phenotype in embryos of the indicated genotypes. Scale bar represents 50µm.

Supplemental References

Comber, K., S. Huelsmann, I. Evans, B.J. Sanchez-Sanchez, A. Chalmers, R. Reuter, W. Wood, and M.D. Martin-Bermudo. 2013. A dual role for the betaPS integrin myospheroid in mediating Drosophila embryonic macrophage migration. *J Cell Sci*. 126:3475-3484.
