# Supplementary material for: Drosophila Embryonic Hemocytes Produce Laminins to Strengthen Migratory Response
Source: Cell Rep. 2017 Nov 7;21(6):1461–70. doi: 10.1016/j.celrep.2017.10.047 (PMC5695906; doi:10.1016/j.celrep.2017.10.047)
Supplement: Document S2. Article plus Supplemental Information [file mmc16.pdf]

Cell Reports

Report

Drosophila Embryonic Hemocytes Produce Laminins to Strengthen Migratory Response

Graphical Abstract

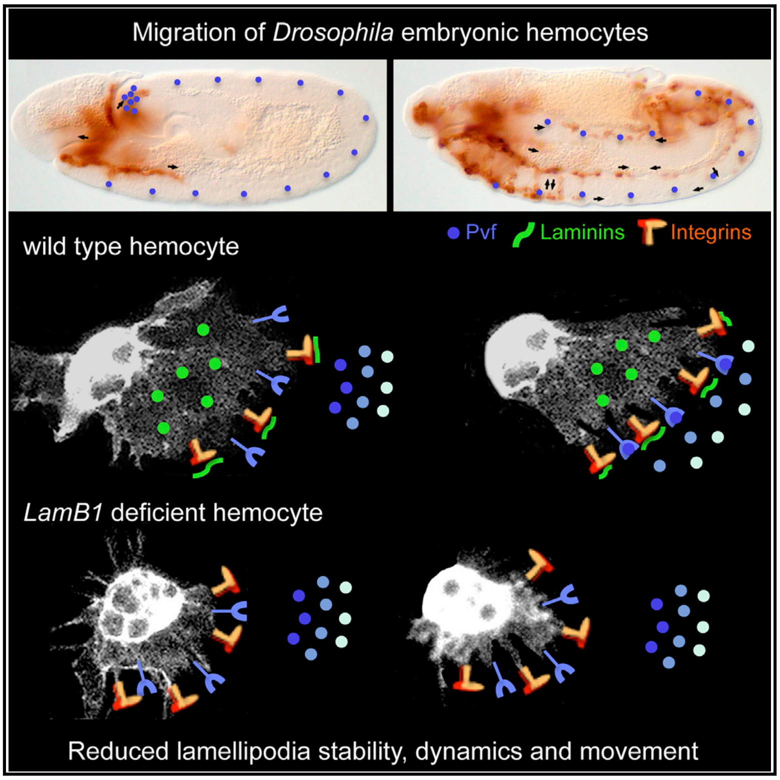

Highlights

- *Drosophila* embryonic hemocytes use autocrine-produced laminins for their migration
- Autocrine laminins regulate lamellipodia dynamics, stability, and persistence
- Rab8 regulates laminin deposition and lamellipodia dynamics in migrating hemocytes
- Laminins deposit in tracks around hemocytes and in a fibrillar mesh over the VNC

Authors

Besaiz J. Sánchez-Sánchez, José M. Urbano, Kate Comber, Anca Dragu, Will Wood, Brian Stramer, María D. Martín-Bermudo

Correspondence

mdmarber@upo.es

In Brief

The role of ECM in regulating developmental cell migration is classically viewed as an exogenous matrix presented to moving cells. In contrast, using the fruit fly, Sánchez-Sánchez et al. show that *Drosophila* embryonic hemocytes use autocrine-produced laminins to regulate lamellipodia stability, dynamics, and persistence, thus reinforcing directional migration.

Sánchez-Sánchez et al., 2017, Cell Reports *21*, 1461–1470
November 7, 2017 © 2017 The Authors.
https://doi.org/10.1016/j.celrep.2017.10.047

CellPress

Cell Reports
Report

OPEN ACCESS
CellPress

Drosophila Embryonic Hemocytes Produce Laminins to Strengthen Migratory Response

Besaiz J. Sánchez-Sánchez,[1,4] José M. Urbano,[2] Kate Comber,[3] Anca Dragu,[4] Will Wood,[3] Brian Stramer,[4] and María D. Martín-Bermudo[1,5,*]
[1]CABD (CSIC-Universidad Pablo de Olavide-JA), Sevilla 41013, Spain
[2]Department of Physiology, Development and Neuroscience, University of Cambridge, Cambridge CB2 3DY, UK
[3]Department of Cellular and Molecular Medicine, Biomedical Sciences, University of Bristol, Bristol BS8 1TD, UK
[4]Randall Centre for Cell and Molecular Biophysics, King's College London, London SE5 9AP, UK
[5]Lead Contact
*Correspondence: mdmarber@upo.es
https://doi.org/10.1016/j.celrep.2017.10.047

SUMMARY

The most prominent developmental function attributed to the extracellular matrix (ECM) is cell migration. While cells in culture can produce ECM to migrate, the role of ECM in regulating developmental cell migration is classically viewed as an exogenous matrix presented to the moving cells. In contrast to this view, we show here that *Drosophila* embryonic hemocytes deposit their own laminins in streak-like structures to migrate efficiently throughout the embryo. With the help of transplantation experiments, live microscopy, and image quantification, we demonstrate that autocrine-produced laminin regulates hemocyte migration by controlling lamellipodia dynamics, stability, and persistence. Proper laminin deposition is regulated by the RabGTPase Rab8, which is highly expressed and required in hemocytes for lamellipodia dynamics and migration. Our results thus support a model in which, during embryogenesis, the Rab8-regulated autocrine deposition of laminin reinforces directional and effective migration by stabilizing cellular protrusions and strengthening otherwise transient adhesion states.

INTRODUCTION

Cell migration plays a key role in a wide variety of biological phenomena that take place both during embryogenesis and in the adult organism. During embryonic development, there are numerous cases where organ or tissue formation depends upon the migration of primordial cells over large distances. Cell migration is also essential for immune cells to monitor the body and for epithelial cells to heal a wound. This fascinating behavior, which involves a large variety of intricately coordinated and controlled processes in normal cells, becomes destructive and damaging when acquired by cancerous cells. Hence, a better understanding of the molecular mechanisms underlying cell migration would not only further our comprehension about embryogenesis but also help us to understand, or even treat, cancer.

Cellular migration is the coordinated movement of cells over a substratum (Horwitz and Webb, 2003). The substratum, depending on the cellular context, can be either the extracellular matrix (ECM) or the surface of other cells. Among the different ECM components, laminins have been shown to play a key role in the migration of several embryonic cell populations in different species (Brown, 2011). During embryonic development, cells have been proposed to migrate on laminins found in the interstitial spaces and/or the basement membrane (BM) surrounding different tissues. However, experiments in cell culture have suggested that both exogenous and endogenous laminins may contribute to cell migration. This is the case for a variety of tumor cells, including melanomas and gliomas. Thus, in addition to migrating on laminins present in the BM of target tissues, tumor cells might also migrate on laminins produced by themselves (Ishikawa et al., 2014; Kawataki et al., 2007; Oikawa et al., 2011). Strikingly, the use of produced laminins during cell migration has not been demonstrated in embryos or adults.

Drosophila embryonic hemocytes are an ideal system to study the role of secreted laminins on cell migration. They are major producers of ECM components and perform a stereotyped migration through the embryo. They derive from the head mesoderm, and the majority become migratory and differentiate into macrophages (Lebestky et al., 2000). At the end of stage 10, embryonic hemocytes initiate their migration throughout the embryo following three major routes, toward the tail, beneath the amnioserosa, and along the ventral nerve cord (VNC) (Tepass et al., 1994). Hemocyte migration along the VNC is required for proper ECM deposition around this tissue (Olofsson and Page, 2005) and its correct development (Evans et al., 2010). Our preliminary analysis of the role of laminins during embryogenesis has shown that hemocytes fail to migrate along the VNC in laminin mutant embryos (Urbano et al., 2009). Thus, as ECM deposition around the VNC requires hemocyte migration and hemocytes need ECM components to move along this path, it is tempting to speculate that hemocytes deposit their own ECM to migrate. If this were the case, it would imply that embryonic migratory cells could behave in an analogous manner to tumor cells in their ability to use ECM component in an autocrine function for their migration. Here, we sought to investigate this by analyzing the role of laminin secretion during embryonic hemocyte migration.

Laminins are trimeric glycoproteins formed from three distinct subunits: α, β, and γ. Members of the laminin family are highly

This is an open access article under the CC BY license (http://creativecommons.org/licenses/by/4.0/).

conserved. While in humans there are 15 different laminin trimers, *Drosophila* only contains two, which possess different α subunits, encoded by the genes wing blister and LanA, but share the same β and γ subunits, encoded by the genes LanB1 and LanB2, respectively. Isolation of LanB1 and LanB2 mutations in *Drosophila* has allowed the analyses of the effects of a complete loss of laminin function during embryogenesis (Urbano et al., 2009; Wolfstetter and Holz, 2012). As mentioned above, our initial analysis on laminin function in hemocyte development has shown that their migration along the VNC is compromised in lanB1 mutant embryos (Urbano et al., 2009). Furthermore, integrins, major laminin receptors, are also required for proper hemocyte migration in the *Drosophila* embryo (Comber et al., 2013).

By combining live imaging with transplantation experiments and quantification analysis, we show that laminins are required for all steps of hemocyte migration throughout the embryo. We further demonstrate that autocrine-produced laminin enhance hemocyte migration by regulating lamellipodia dynamics and stability. Finally, our results uncover a function for the Rab8 RabGTPase in hemocyte migration, as Rab8 is required for proper laminin secretion and lamellipodia dynamics. Based on these results, we propose a model in which autocrine laminin deposition by hemocytes, regulated by Rab8, would reinforce directionally persistent and effective migration by stabilizing protrusions and strengthening otherwise transient adhesion states.

RESULTS

Laminins Regulate Hemocyte Migration over the VNC

Migrating hemocytes produce both laminins and collagens (Mirre et al., 1988; Montell and Goodman, 1989; Yasothornsrikul et al., 1997). In a preliminary analysis of the role of laminins during *Drosophila* embryogenesis, we showed that hemocytes within embryos homozygous for a deficiency that removes the *LanB1* gene, hereafter referred to as *LanB1* embryos, failed to migrate along the VNC. Quantification of the migration phenotype (Figure S1) showed that while most wild-type embryos show no defect (83.3%, n = 48; Figure S1), hemocyte migration was disrupted in 98.3% of *LanB1* embryos (n = 52; Figure S1). To investigate in greater detail the role of laminins in hemocyte migration, we performed live imaging of embryos expressing UAS-GFPMoesin within hemocytes, using the *srpH*Gal4 driver (*srpH* > GFPMoe; Wood et al., 2006).

In wild-type embryos, hemocyte migration occurs in several phases (Brückner et al., 2004; Cho et al., 2002; Tepass et al., 1994). Around stage 10, one group of hemocytes enter into the posterior end of the germband, surround the hindgut, and migrate to the posterior end of the developing VNC. We found that hemocytes from *LanB1* embryos (n = 33) surrounded the hindgut at a slower speed (0.54 ± 0.08 μm/min) than hemocytes from control embryos (n = 31, 1.7 ± 0.15 μm/min; Figures 1A–1G; Movie S1). In the next phase of migration (stage 12), hemocytes from the anterior and posterior ends of the embryo move toward one another along both sides of the VNC (Brückner et al., 2004; Cho et al., 2002; Tepass et al., 1994). Thus, by stage 13, hemocytes form a single band covering the entire VNC immediately beneath the epidermis (Movie S2). In contrast, we found that in 98.3% of stage 13 *LanB1* embryos, hemocytes do not cover completely the VNC (Figure S1B). Our *in vivo* analysis revealed that hemocytes from *LanB1* embryos (n = 30) moved on both sides of the VNC at a slower speed than hemocytes from control embryos (n = 32), although migration was more affected on the ventral (1.73 ± 0.11 μm/min versus 3.06 ± 0.12 μm/min) than on the dorsal side (2.65 ± 0.14 μm/min versus 2.97 ± 0.07 μm/min; see below; Figures 1H and 1I; Movie S2).

We have previously shown that hemocytes fail to progress along the midline in integrin mutant embryos. This was partially due to a failure in the separation of the VNC from the overlying epithelium. We thus proposed that the role of integrins in the VNC was to regulate proper assembly of ECM components, which in turn could be required for a correct separation of the VNC (Comber et al., 2013). Indeed, using dye injections (Evans et al., 2010), we found that in contrast to stage 15 control embryos, in which the dye permeates along the length of the embryo (Figure S2A; n = 17), dye spreading in *LanB1* embryos is restricted, indicating areas where the epithelium failed to detach from the VNC (Figure S2B; n = 15). This could explain why migration speed was more affected on the ventral side than on the dorsal side in *LanB1* embryos compared to controls (Figures 1H and 1I).

Together, these results reveal hemocyte specific and environmental requirements for laminins during hemocyte migration.

Hemocytes Require Laminins for Lateral, Random, and Inflammatory Migrations

At stage 13, hemocytes from wild-type embryos undergo a segmented and highly directional lateral migration away from the ventral midline, leading to the formation of three rows of hemocytes covering the ventral side of the VNC (Figure S3A; Movie S3) (Wood et al., 2006). We found there was a reduction in the number and speed of hemocytes migrating laterally in *LanB1* embryos (n = 72 and n = 30, respectively) compared to controls (n = 102 and n = 28, respectively) (Figures S3A–S3D; Movie S3).

At stage 15, wild-type hemocytes move locally in a random manner and undergo contact repulsion, which is key to maintain their even distribution (Movie S4; Stramer et al., 2010; Wood et al., 2006). Hemocytes from *LanB1* embryos (n = 42) moved locally at a slightly but significantly slower speed (2.31 ± 0.09 μm/min) than control cells (n = 41) (2.76 ± 0.15 μm/min) (Figures S3E–S3G; Movie S4). In addition, while the average time that control hemocytes remained in contact was ~5 min (n = 72), hemocytes from *LanB1* embryos (n = 58) remained in contact for prolonged periods (7.5 min) (Figures S3H–S3J).

Finally, we analyzed laminin requirements in hemocytes during their inflammatory migration to wounds. To do this, we used laser ablation to create epithelial wounds in the anterior trunk of the embryo, in areas occupied with hemocytes. While hemocytes from *LanB1* embryos were competent to respond to an inflammatory cue (Figure S4), there was a significant reduction in the speed and number of hemocytes recruited to wounds at the early stages of this inflammatory migration (Figure S4C). Together, these results show that laminins are required for both directed and random migrations.

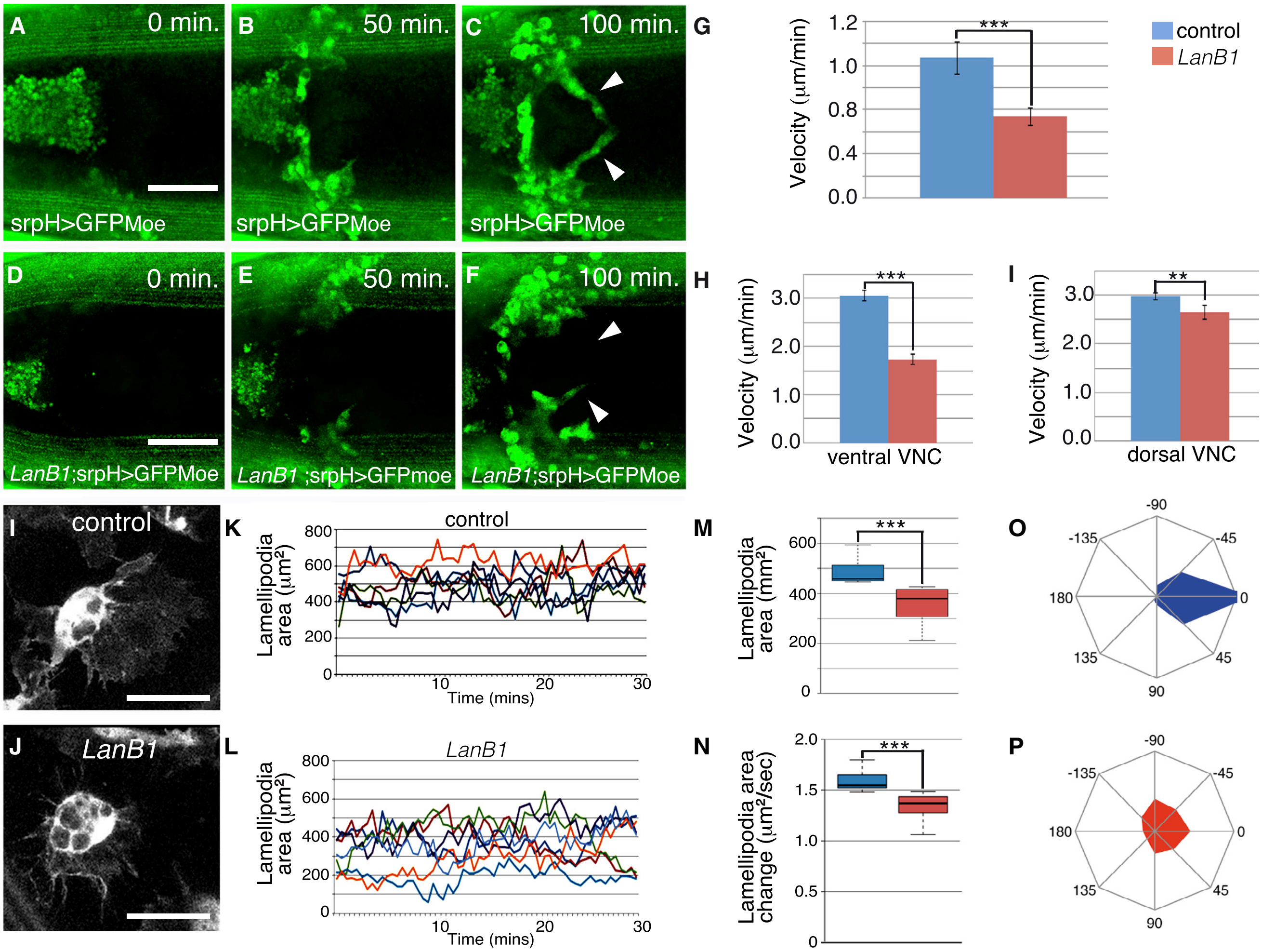

Figure 1. Laminins Are Required for Proper Hemocyte Migration around the Hindgut and Over the VNC and for Lamellipodia Dynamics and Persistence

(A–F) Stills taken from live imaging of GFPMoe-expressing hemocytes migrating around the hindgut in control (A–C) and *LanB1* mutant embryos (D–F).

(G) Tracking individual hemocytes reveals a significant decrease in the velocity of hemocytes from *LanB1* mutant embryos ($p < 0.001$).

(H and I) Graph showing a significant decrease in the velocity of hemocytes from *LanB1* embryos compared to control on both the (H) ventral ($p < 0.0001$) and (I) dorsal sides of the VNC ($p < 0.01$).

(I and J) Hemocytes from control (I) and *LanB1* (J) embryos.

(K and L) Graphs showing the lamellipodial area of (K) control (n = 6) and (L) *LanB1* (n = 7) hemocytes measured at 30-s intervals over a 30-min time period.

(M and N) Average lamellipodial area (M) and lamellipodial area change (N) per hemocyte from control and *LanB1* embryos (n = 6 and 7 hemocytes from 3 different embryos per genotype, respectively).

(O and P) Radial diagrams illustrating lamellipodia distribution in control (O) and *LanB1* embryos (P).

Scale bars represent 50 μm (A–F) and 20 μm (I and J).

Laminins Regulate Proper Lamellipodia Formation and Dynamics

We next decided to test whether alterations in the migratory behavior of *LanB1* hemocytes could be the result of aberrant lamellipodia dynamics. To this end, we analyzed lamellipodia area over time in hemocytes from control and *LanB1* embryos during the phase of random migration. Our results revealed that both lamellipodia dynamics and area were affected in *LanB1* embryos (Figure 1I–1N). The fact that the lamellipodia area was significantly reduced (30%) in hemocytes from mutant embryos (353 ± 6 μm^2, n = 49) compared to controls (506 ± 5 μm^2, n = 47) (Figure 1I–1N) suggests that laminins might be required for the cell-adhesion events that take place during cell migration.

Formation and stabilization of lamellipodia plays a critical role in achieving directionally persistent migration in cell culture (reviewed in Petrie et al., 2009). Our *in vivo* analysis reveals that migrating hemocytes from control embryos seem to sustain over time a relatively constant orientation of lamellipodia protrusion in the direction of the movement. To quantify this, we analyzed lamellipodia distribution over time during random migration (Movie S4). We found that hemocytes

OPEN
ACCESS
CellPress

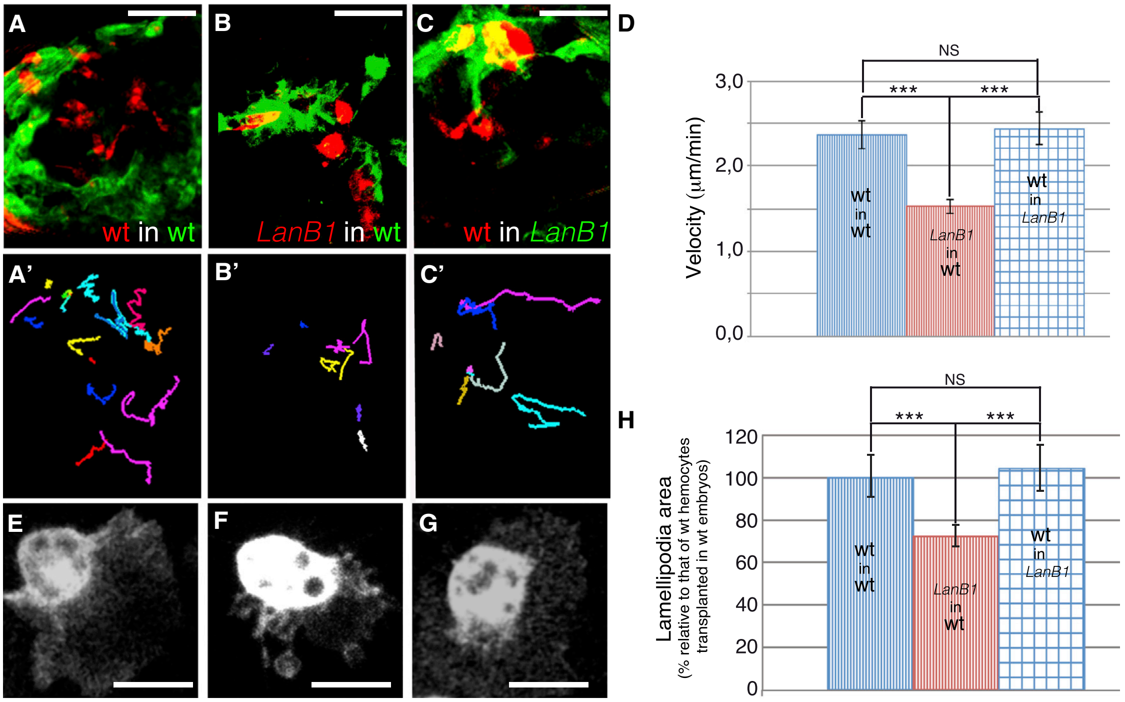

Figure 2. Hemocytes Provide Autocrine Laminins as a Substrate for Their Movement

(A–C) Still images taken from live imaging of transplanted hemocytes from (A) control embryos (red) into control embryos (green), (B) *LanB1* embryos (red) into control embryos (green), and (C) control embryos (red) into *LanB1* embryos (green).

(A′–C′) Tracking analysis of (A)–(C), respectively.

(D) Quantification of hemocyte migration speed in the transplanted embryos of the indicated genotypes.

(E–G) Hemocytes from control embryos in control (E) and LanB1 mutant embryos (G) and hemocytes from *LanB1* in control embryos (F).

(H) Graph showing lamellipodia area of transplanted hemocytes relative to controls (hemocytes from control embryos transplanted in control embryos).

Scale bars represent 30 μm (A–C) and 20 μm (E–G).

from control embryos extend and maintain lamellipodia protrusion preferentially in the direction of migration (Figure 1O). This persistence was reduced in hemocytes from *LanB1* embryos (Figure 1P).

Altogether, these results strongly suggest that laminins support directional migration by regulating lamellipodia formation, dynamics, and persistence.

Hemocytes Provide Laminins in an Autocrine Fashion for Their Migration

During *Drosophila* embryogenesis, several cell populations produce laminins, including hemocytes, fat body, and some neuronal cells (Kusche-Gullberg et al., 1992; Montell and Goodman, 1989). Hence, there are multiple sources of laminins hemocytes could use as a substrate during their migration throughout the embryo, including an autonomous one produced by hemocytes themselves, an exogenous one, or both. To test the contribution of the laminins produced autonomously by hemocytes, we used two strategies. First, we specifically expressed RNAi constructs against the *LanB1* or *LanB2* genes in either hemocytes (n = 108; Figures S5B and S5D; data not shown) or in glia and neural progenitor cells. We found that while the expression of *LanB1* or *LanB2* RNAis in hemocytes resulted in migration defects similar to those observed in *LanB1* embryos, their expression in neuronal cells only caused subtle defects (Figures S5C and S5D). Second, we developed a method for transplanting hemocytes from the head mesoderm of donor embryos into the VNC of host embryos. To distinguish donor from host hemocytes, donors were labeled using mChMoe while hosts were marked with GFPMoe (see Experimental Procedures). We performed a number of fluorescently labeled hemocyte transplantations, including control hemocytes into control embryos (n = 10), control hemocytes into *LanB1* embryos (n = 10), and *LanB1* hemocytes into control embryos (n = 12). We then analyzed their migration *in vivo* and quantified lamellipodia areas of the different hemocyte types (Figure 2; Movies S5, S6, and S7). We found that the migration speed of control hemocytes transplanted into control embryos (2.44 ± 0.17 μm/min) was similar to that of control hemocytes transplanted into *LanB1* embryos (2.37 ± 0.08 μm/min). However, the speed of *LanB1* hemocytes transplanted into control embryos was significantly slower (1.52 ± 0.08 μm/min; Figure 2D). Furthermore, we found that the lamellipodia area of hemocytes was 27.6% smaller in *LanB1* embryos transplanted in control embryos than in hemocytes from control embryos transplanted in control or *LanB1* embryos (Figures 2E–2H).

Overall, these results implicate autocrine-produced laminin in the regulation of hemocyte lamellipodia dynamics and thus in the control of their directional migration.

OPEN ACCESS
CellPress

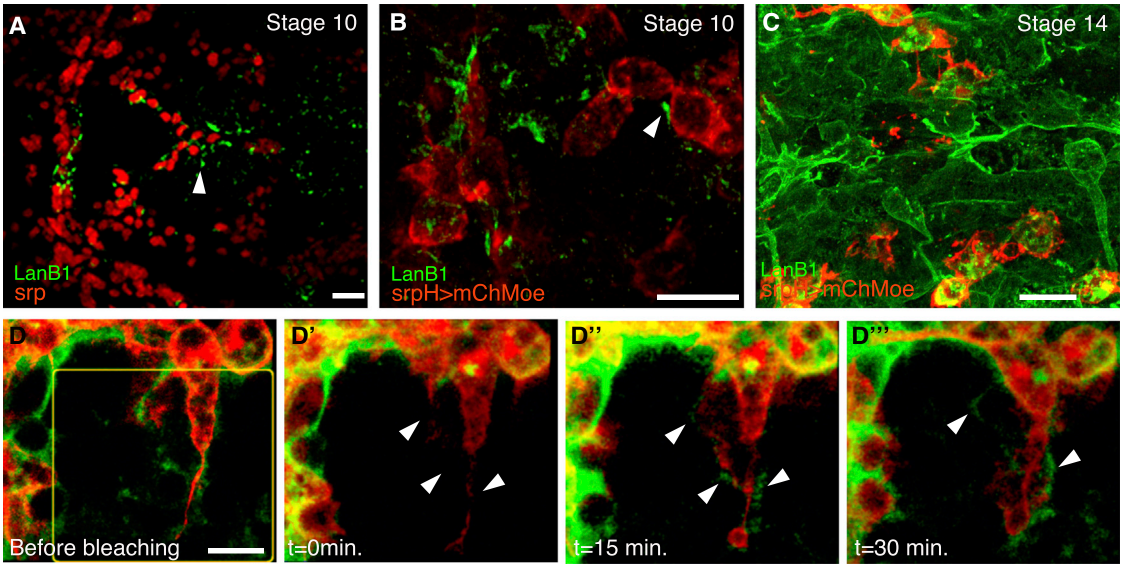

Figure 3. LanB1-GFP Distribution around Hemocytes and the VNC

(A) Dorsal view of a fixed stage 10 control embryo expressing a LanB1-GFP fosmid stained with anti-GFP (green) and anti-Srp (red) antibodies. LanB1-GFP can be found around migrating hemocytes (arrowhead).

(B–D) Still images taken from live LanB1-GFP; srpH > mChMoe embryos. Hemocytes entering the tail in stage 10 embryos (B). LanB1-GFP can be found in track-like arrays around hemocytes (arrowhead). Dorsal view of a stage 14 embryo showing LanB1-GFP decorating the BM surrounding the VNC (C). Representative images of hemocytes over the VNC of a stage 14 embryo during a FRAP experiment (D).

Scale bars represent 20 μm (A), 15 μm (B and C), and 10 μm (D).

Laminins Assemble in Different Patterns during Embryogenesis

Cell culture experiments have shown that laminins can be assembled in diverse patterns (Kariya et al., 2012). Furthermore, it has been speculated that these different laminin patterns might specify cell behaviors (reviewed in Hamill et al., 2009; Sehgal et al., 2006).

In stage 11 and 12 *Drosophila* embryos, laminins have been detected in the mesodermal layer, cephalic mesoderm, hemocytes, and fat body (Kusche-Gullberg et al., 1992; Montell and Goodman, 1989). Upon germband retraction, laminins were also found as part of the dorsal BM overlying the VNC (Montell and Goodman, 1989). These studies were performed using antibodies against the LanB1 and LanB2 subunits and conventional light microscopy in fixed tissue. Although informative, these studies did not allow the detection of putative specific patterns in which laminins might assemble *in vivo*. To tackle this issue, we used a fosmid containing a GFP-tagged *LanB1* gene (Sarov et al., 2016) and high-resolution methods for *in vivo* imaging (see Experimental Procedures). Our results show that hemocytes undergoing either developmental or inflammatory migrations contain laminin-rich punctae (Figure 3A; Movie S8). In addition, laminin is also found around hemocytes assembled in track-like arrays (Figure 3B; Movie S9). To determine whether these trails of laminins around migrating hemocytes are deposited by hemocytes themselves, we examined fluorescence recovery after photobleaching (FRAP) of LanB1-GFP in a region containing hemocytes undergoing lateral migration (Figures 3D–3D″; Movie S10). Deposition of LanB1-GFP was detected around hemocytes in the photobleached area 2 min after bleaching (Figures 3D and 3E; Movie S10). Interestingly, LanB1-GFP deposition in trails was particularly prominent around extended lamellipodia (Movie S10, arrows). These results suggest that hemocytes assemble trails of laminins that could be used as a substratum over which they move. Next, we analyzed in more detail LanB1-GFP distribution around the VNC by performing high-resolution analysis and 3D imaging of live dissected stage 14 embryos (see Experimental Procedures). We found that LanB1-GFP was organized as a fibrillar mesh surrounding the VNC (Figure 3C; Movie S11).

In sum, these results show that laminins can be assembled and/or deposited in different patterns throughout embryogenesis.

Rab8 Is Required for Proper Hemocyte Migration

The Rab family of GTPases, and in particular Rab8 and Rab10, have been shown to regulate secretion of ECM components. In addition, cell culture studies have revealed important functions for Rab8 in regulating cell migration (Hattula et al., 2006). Thus, we next tested whether these Rabs play any role during hemocyte migration. First, we investigated if they were expressed in hemocytes as they migrate. To this end, we used transgenic flies in which the coding sequence for these Rabs has been N-terminally tagged, by homologous recombination, with YFP, hereafter referred as Rab8YFP and Rab10YFP (Dunst et al., 2015). We observed that while Rab8YFP was found at high levels in cytoplasmic puncta in migrating hemocytes, Rab10YFP was barely detected (Figures 4A and 4B). Furthermore, as it happens in *Drosophila* follicle (Devergne et al., 2017) and embryonic epithelial cells (Mavor et al., 2016), a proportion of Rab8YFP positive puncta co-express the *cis*-Golgi marker GM130 (Movie S12),

OPEN
ACCESS
CellPress

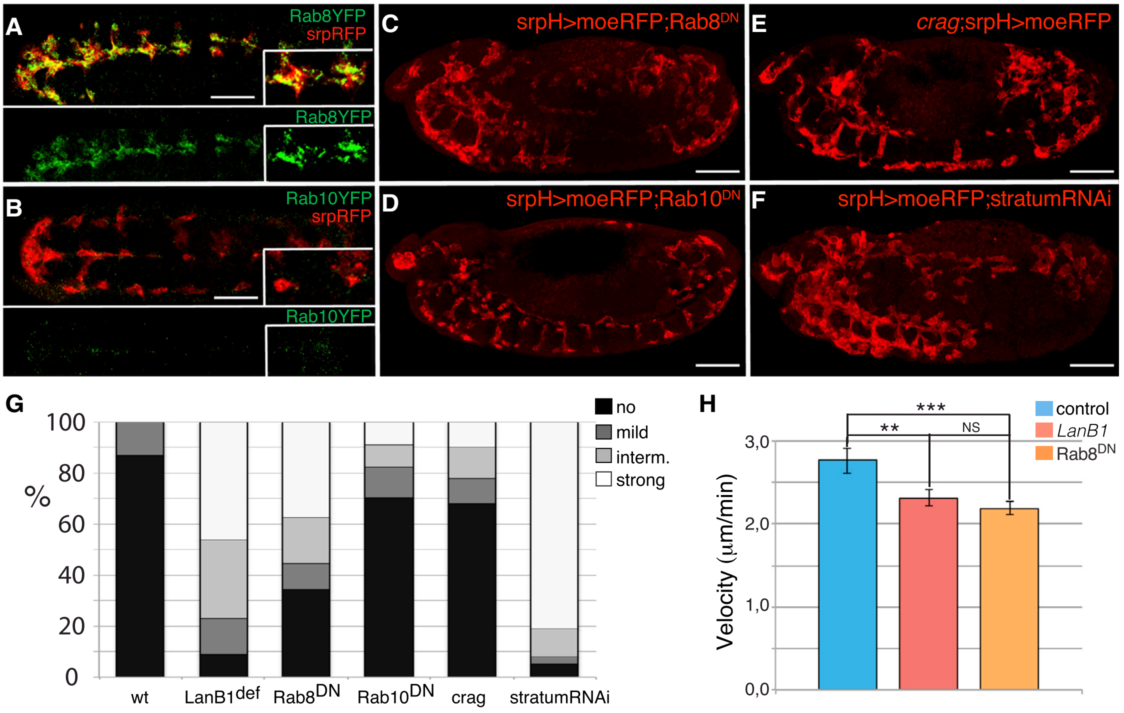

Figure 4. The RabGTPase Rab8 Is Required for Proper Hemocyte Migration

(A and B) Ventral view of fixed stage 13 embryos expressing mChMoe in hemocytes (srpH > mChMoe) and Rab8YFP (A) or Rab10YFP (B) stained with anti-RFP (red) and anti-GFP (green) antibodies.

(C–F) Lateral view of fixed stage 13 embryos expressing UAS-mChMoe and UAS-Rab8^{DN} (C), UAS-Rab10^{DN} (D), or a UAS-RNAi (F) against *stratum* under the control of the *srpH-Gal4* driver and staining with an anti-RFP antibody. Stage 13 *crag* mutant embryo expressing mChMoe in hemocytes (E).

(G) Quantification of hemocyte migration phenotype over the VNC in embryos of the indicated genotype.

(H) Quantification of migration speed in hemocytes expressing Rab8^{DN} compared to hemocytes from control and *LanB1* embryos during random migration. The statistical significance of differences was assessed with a t test; ***$p < 0.0001$ and **$p < 0.005$.

Scale bars represent 50 μm.

consistent with the known role of Rab8 in regulating vesicle trafficking from the Golgi to the plasma membrane (Wandinger-Ness and Zerial, 2014).

Accordingly, expression of a dominant-negative form of Rab8 (Rab8^{DN}; Figure 4C; n = 110) (Zhang et al., 2007), or an RNAi against its recently identified GEF *stratum* (Figure 4F; n = 92) (Devergne et al., 2017), specifically in hemocytes impaired their migration (Figure 4G). *In vivo* analysis of random migration revealed that similar to hemocytes from LanB1 embryos, hemocytes expressing Rab8^{DN} move at a slower speed (2.19 ± 0.08 μm/min) than controls (2.75 ± 0.15 μm/min; Figure 4H; Movie S13). In contrast, hemocytes expressing a dominant-negative form of Rab10 (Rab10^{DN}; n = 68) (Zhang et al., 2007) or mutant for *crag* (n = 50) showed minor migratory defects (Figures 4D, 4E, and 4G).

In an attempt to test whether these Rabs were required for proper laminin secretion, we quantified LanB1-GFP levels around the VNC in stage 13 embryos expressing Rab8^{DN} specifically in hemocytes and compared them to those found in control embryos. Because hemocyte migration is impaired in the experimental condition, there were areas of the VNC packed with hemocytes where LanB1-GFP levels could be higher simply due to the increased number of hemocytes. To avoid this, we only analyzed areas with similar number of hemocytes in control and experimental embryos (Figures 5A and 5B). We found that LanB1-GFP levels in the VNC were higher in experimental embryos than in control embryos (28.07 ± 3.80 versus 17.65 ± 2.71; Figures 5A–5C). In addition, high-resolution analysis and 3D imaging of hemocytes entering the tail revealed that levels of laminins outside of hemocytes were higher and more uniformly distributed around Rab8^{DN}-expressing hemocytes compared to controls (Movie S14; compare with Movie S9).

We have shown here that proper lamellipodia formation and dynamics require laminins produced autonomously by hemocytes. As laminin levels and distribution are affected in Rab8^{DN}-expressing hemocytes, we tested whether lamellipodia dynamics were altered in these mutant hemocytes. Indeed, we found that similar to the elimination of laminins, expression of Rab8^{DN} in hemocytes results in smaller (314.45 ± 6 μm^2 versus 504 ± 7 μm^2) and less stable and persistent lamellipodia (Figures 5D–5I).

DISCUSSION

Laminins are expressed in tumor-target tissues as well as in many tumor cells, including glioma, breast cancer, and

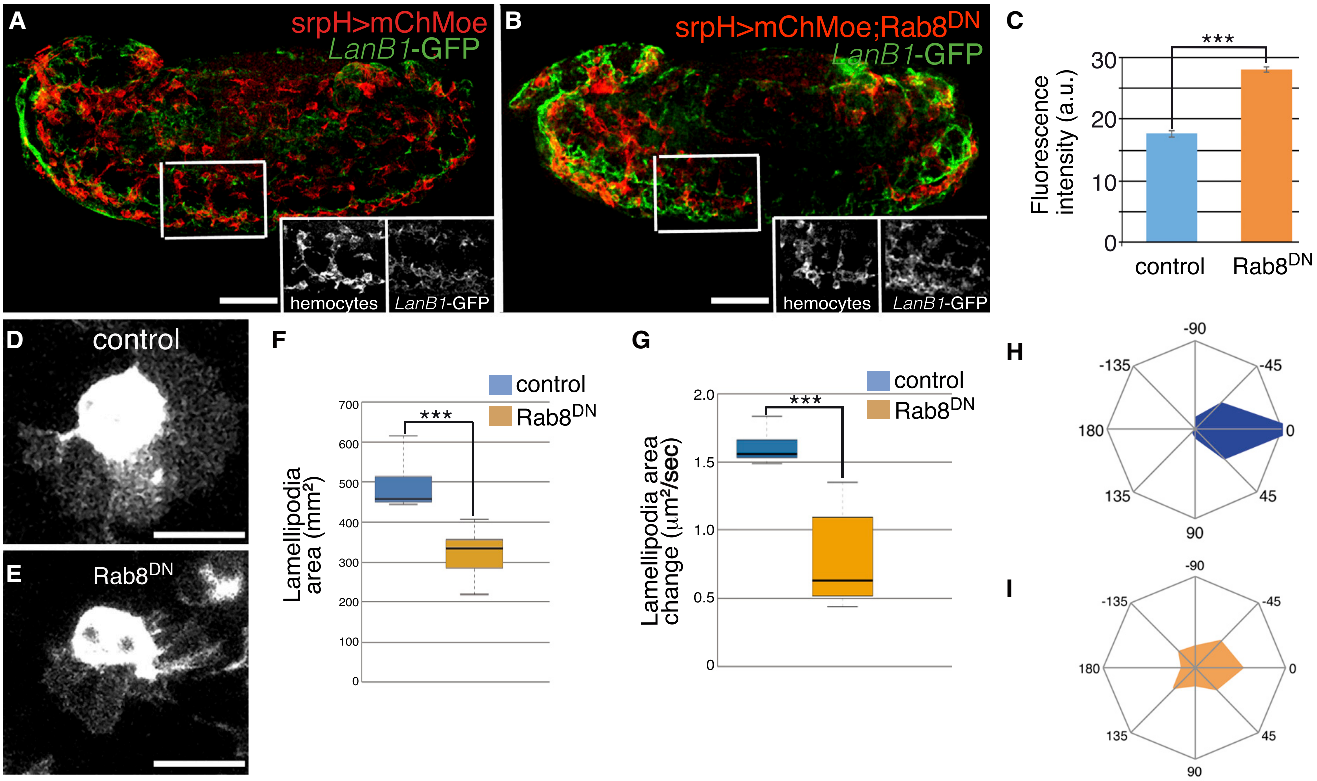

Figure 5. Rab8 Regulates LanB1-GFP Secretion and Lamellipodia Dynamics

(A) Lateral view of fixed stage 15 embryos expressing mChMoe in hemocytes (srpH > mChMoe) and LanB1-GFP stained with anti-RFP (red) and anti-GFP (green) antibodies.

(B) Role of Rab8 on regulating laminin secretion was assessed by co-expressing UAS-Rab8^{DN}.

(C) Quantification of LanB1-GFP intensity in the VNC of embryos expressing UAS-Rab8^{DN} in hemocytes.

(D and E) Control (D) and Rab8^{DN}-expressing (E) hemocytes.

(F–I) Average lamellipodial area (F), lamellipodial area change (G), and radial diagrams illustrating the distribution of lamellipodia in control (H) and Rab8^{DN}-expressing hemocytes (I).

Scale bars represent 50 μm (A and B) and 20 μm (D and E).

malignant melanoma cells. This has led to the proposition that both exogenous and endogenous laminins may contribute to tumor dissemination (Chia et al., 2007; Ishikawa et al., 2014; Kawataki et al., 2007; Oikawa et al., 2011). Single-cell assays have shown that human keratinocytes also deposit laminin 5 to promote persistent and linear migration in the absence of a chemotactic gradient (Frank and Carter, 2004). Here, we show that *Drosophila* embryonic hemocytes use both exogenous and endogenous laminins for their migration over the VNC. Furthermore, our transplantation experiments and our results showing that migration defects are stronger when laminins are reduced in hemocytes than when they are decreased in the VNC demonstrate that endogenous laminins are more relevant to hemocyte migration than exogenous ones. Our discovery that laminins regulate lamellipodia stability and dynamics suggests that by using endogenous laminins, hemocytes could stabilize cellular protrusions locally, thus reinforcing directional migration. In fact, culture assays and xenograft of fibrosarcoma HT1080 cells have shown that fibronectin is endocytosed and re-secreted from a late endosomal/lysosomal compartment to provide an autocrine ECM substrate to enhance directional migration (Sung and Weaver, 2011; Sung et al., 2015). Local concentrations of ECM molecules via secretion may facilitate integrin clustering and/or activity, leading to accelerated migration. In addition, many ECM components have binding sites for growth factors (Kim et al., 2011). Thus, local levels of ECM molecules could also allow high confined concentrations of growth factors, which in turn would enhance their activity.

The mechanisms underlying laminin secretion remain largely unknown. Recent experiments in *Drosophila* have shown that downregulation of Rab10 and its GEF Crag results in aberrant secretion of BM components, including laminins, at the apical side of follicle cells (Denef et al., 2008; Lerner et al., 2013). Here, we find that Rab8 is required in migrating embryonic hemocytes for proper laminin secretion and migration. Thus, we propose that Rab8 might serve a function in hemocytes similar to that perform by Rab10 in epithelial follicle cells (i.e., the control of proper secretion of ECM components). This in the context of epithelial cells is essential for correct morphogenesis while in the context of migrating cells is key for efficient and directional movement. In fact, cell culture experiments have demonstrated that appropriate levels of ECM ligands are essential for correct

cell movement, with a maximal velocity at middle levels of substrate and a block in migration at higher substrate concentrations (Palecek et al., 1997). Our results also show that, as is the case in cell culture experiments (Hattula et al., 2006), Rab8 regulates lamellipodia dynamics and stability during developmental cell migration. Because laminins are also required for proper lamellipodia dynamics, the role of Rab8 in regulating lamellipodia dynamics could be in part mediated via its ability to control proper laminin secretion. However, besides a role in regulating secretion of ECM proteins, the Rab GTPases have also been involved in controlling membrane trafficking routes as well as endocytosis and trafficking of growth factor receptors, integrins, and RhoGTPases during cell migration (Bridgewater et al., 2012; Porther and Barbieri, 2015). In the future, it will be interesting to determine the contribution of the different putative roles of Rab8 to cell migration during development.

Cell culture experiments have shown that despite their relatively uniform structure, laminins are deposited into diverse patterns in the ECM depending on the cell type (Kariya et al., 2012). Furthermore, it has been proposed that differences in laminin patterns might be essential in specifying cellular behaviors. Keratinocytes provide an interesting example of how laminin deposition patterns might affect matrix function. In non-migratory human keratinocytes, laminin-332 appears in a rosette-like pattern, whereas actively migrating keratinocytes in culture assemble trails of laminin-332 over which they move (Hamill et al., 2009; Sehgal et al., 2006). Lung alveolar epithelial cells organize a laminin-331 matrix into fibrils, which is used to transmit mechanosignals in the form of stretch (Jones et al., 2005). Similarly, a polarized fibrillar BM has been shown to constrain tissue shape during egg chamber elongation in *Drosophila* (Frydman and Spradling, 2001; Haigo and Bilder, 2011). Our *in vivo* analysis of laminin expression reveals that laminins are organized at least in two different patterns in the *Drosophila* embryo: in track-like arrays surrounding migrating hemocytes and, at the end of embryogenesis, in a fibrillar mesh surrounding the VNC. We would like to propose that these distinct ways in which laminins are assembled and/or deposited might serve distinct laminin functions. Thus, while the role of laminins deposited by hemocytes might be to promote a migratory behavior, laminins deposited in the BM of the VNC could be required to transmit forces necessary for VNC condensation. The former is supported by our results showing that reducing the levels of laminins specifically in hemocytes affects their migration. The latter is supported by results showing that reducing ECM components around the VNC, by preventing hemocyte migration, leads to VNC condensation defects (Olofsson and Page, 2005) and by our own results showing that VNC condensation is affected in *LanB1* embryos (Urbano et al., 2009). In the future, it will be interesting to assess to what extent differences in laminin assembly and/or deposition specify cell behaviors and to dissect the mechanisms underlying laminin-deposition and/or assembly patterns.

Laminins have been widely reported to promote cancer cell migration and tumor progression. They have been recently termed as "oncolaminins" because (1) they are expressed in tumor cells correlating with malignancy, (2) their expression is positively regulated by oncogenic transcription factors, and (3) they interact with integrins, which was previously implicated in tumor invasion and progression. Accordingly, human antibodies to specific domains of the laminin proteins inhibit tumor growth and metastasis of melanoma (Mills et al., 2002). In addition, as we have shown here for embryonic hemocytes, tumor cell-produced laminins contribute to tumor migration and dissemination (Chia et al., 2007; Ishikawa et al., 2014; Kawataki et al., 2007; Oikawa et al., 2011). Thus, we can exploit the advantages offered by *Drosophila* embryonic hemocytes to provide novel insights into the molecular and cellular mechanism by which laminins control robust migratory responses both during development and in physiological and pathological conditions, such as wound healing and cancer.

EXPERIMENTAL PROCEDURES

Drosophila Strains

Flies were raised at 25°C. Embryos were collected from laying cages kept overnight at 25°C. For RNAi experiments, laying cages were kept overnight at 29°C. The following stocks were used: *w; LanB1^{Def}/CyO* (Urbano et al., 2009), *ywcrag^{A}*{neoFRT}19A/FM7c (Bloomington *Drosophila* Stock Center), srp-HemoGAL4 (Brückner et al., 2004), *elav-GAL4* (Berger et al., 2007), UASp-YFP.Rab8.T22N and UASp-YFP.Rab10.T23N (Zhang et al., 2007), Rab8^{YFP} and Rab10^{YFP} (Dunst et al., 2015), UAS-RNAi LanB2 and UAS-RNAi LanB1(Vienna *Drosophila* Resource Center [VDRC]), and UAS-stratum (Bloomington).

For time-lapse imaging, *serpent-HemoGAL4* (*srpH*) (Brückner et al., 2004) was used to drive hemocyte-specific expression of the UAS constructs UAS-GFP-Moesin (Dutta et al., 2002) and UAS-mCherry-Moesin (a gift from Paul Martin, University of Bristol).

Histochemistry

Antibody staining of embryos was performed using standard procedures. We used the following primary antibodies: rat anti-RFP (1:200, Chromotek), rabbit anti-LanB1 (1:500, Abcam), chicken anti-GFP (1:500, Abcam), rabbit anti-Srp (1:5,000; Riechman et al., 1998), mouse anti-CD2 (1:7, DHSB, Iowa), and rabbit anti-GM130 (1:200, Abcam). Fluorescence-conjugated antibodies used were Alexa Fluor 488, Alex Fluor 568, Alexa Fluor 555 (Life Technologies). For non-fluorescent staining, embryos were incubated in biotinylated secondary antibodies followed by incubation with Elite ABC complex (Vector Laboratories) and revealed with 3,3′-diaminobenzidine (DAB; Gibco). Images were collected with a Leica SP2-AOBS, a Leica SP5-MP-AOBS confocal microscope, and a Zeiss LSM 880.

For live imaging of LanB1-GFP around the VNC, we used a protocol based on the one described by Lee et al. (2009). In summary, dechorionated embryos were placed dorsal side down on a small rectangular slab of agar. The embryos were then transferred to a slide covered with a double-sided sticky tape. 1× PBS was immediately added over the embryos, and a cut was made along the dorsal midline using an insect pin/needle. Then, the flaps of the body wall were gently pasted down onto the slide, avoiding stretching or removing material from the surface. PBS was then removed and replaced with fluorescent mounting medium (Vectashield, Vector Laboratories), and live imaging was performed.

Image Processing and Analysis

Cell tracking and cell area measurements were performed using ImageJ (NIH) and Imaris (Bitplane). Co-localization analysis was done using Imaris. Integrated intensity of Rab8YFP and GM130 were quantified for manually selected hemocytes. The background value taken from cell-free regions was subtracted from all data series. Measurements of lamellipodia distribution were done manually as described previously (Fernández-Espartero et al., 2013). In summary, a line was drawn from the center of the hemocyte body to the tip of the lamellipodia. To analyze lamellipodia orientation, the angle of this line relative to the x axis was calculated. Graphs and statistical analysis was carried out using Prism for Mac (GraphPad). Unless otherwise stated, hemocyte migratory behavior was analyzed using images acquired from five embryos of each genotype.

Time-Lapse Recording, Wounding, and Dextran Injections

Embryos were prepared and mounted as previously described (Stramer et al., 2010; Wood et al., 2006). Imaging for all steps of hemocyte migration, including migration into the tail, along the VNC, lateral and random, as well as that for contact repulsion of stage 15 embryos, wounding assays, and dextran injections, was performed as previously described (Comber et al., 2013). All live imaging was conducted on a Leica SP5 MP-AOBS confocal microscope, except for wounding assays and dextran injections, which were imaged on a spinning disc confocal microscope (Ultraview; PerkinElmer; Comber et al., 2013).

Transplantation Assay

Embryos were dechorionated as per live imaging. Donor and receptor embryos were mounted in two parallel lines facing each other over a glass slide covered with a double-sided sticky tape. They were dried for 1 min using silica gel and then covered with a drop of Voltalef oil (10S). Using a Narishige micromanipulator, hemocytes from the head mesoderm of donor embryos were sucked and released into the VNC of receptor embryos. To differentiate donor from host hemocytes, donors were labeled using mChMoe, whereas hosts were marked with GFPMoe. After this step, embryos were kept at 25°C for 3 hr to allow healing of the wound generated during the transplantation procedure. To preserve a certain degree of humidity during this time, embryos were placed in a petri dish containing a roll of wet tissue. After this step, embryos were imaged live as described previously (Stramer et al., 2010; Wood et al., 2006).

SUPPLEMENTAL INFORMATION

Supplemental Information includes five figures and fourteen movies and can be found with this article online at https://doi.org/10.1016/j.celrep.2017.10.047.

AUTHOR CONTRIBUTIONS

B. J.S.-S. performed most of the experiments, with contributions from J.M.U., M.D.M.-B., K.C., and A.D. B.S. suggested valuable experiments, and B.S. and W.W. made useful comments on the manuscript. M.D.M.-B. conceived, designed, and wrote the paper.

ACKNOWLEDGMENTS

We thank the Bloomington Stock Center for fly stocks and sharing valuable reagents. We also thank A. González-Reyes for helpful remarks on the manuscript. This work was supported by the Ministerio Español de Ciencia y Tecnología (grants BFU2013-48988-C2-1-P and BFU2016-80797 to M.D.M.-B.) and the Wellcome Trust (grant 090899/Z/09/Z to W.W.). B.J.S.-S. was supported by an FPI studentship from the MCYT, B.S. was supported by grant 107859/Z/15/Z, and K.C. was supported by an MRC doctoral training grant. The institutional support from the Junta de Andalucía to the CABD is acknowledged.

Received: July 12, 2017
Revised: September 5, 2017
Accepted: October 11, 2017
Published: November 7, 2017

REFERENCES

Berger, C., Renner, S., Lüer, K., and Technau, G.M. (2007). The commonly used marker ELAV is transiently expressed in neuroblasts and glial cells in the Drosophila embryonic CNS. Dev. Dyn. *236*, 3562–3568.

Bridgewater, R.E., Norman, J.C., and Caswell, P.T. (2012). Integrin trafficking at a glance. J. Cell Sci. *125*, 3695–3701.

Brown, N.H. (2011). Extracellular matrix in development: insights from mechanisms conserved between invertebrates and vertebrates. Cold Spring Harb. Perspect. Biol. *3*, a005082.

Brückner, K., Kockel, L., Duchek, P., Luque, C.M., Rørth, P., and Perrimon, N. (2004). The PDGF/VEGF receptor controls blood cell survival in Drosophila. Dev. Cell *7*, 73–84.

Chia, J., Kusuma, N., Anderson, R., Parker, B., Bidwell, B., Zamurs, L., Nice, E., and Pouliot, N. (2007). Evidence for a role of tumor-derived laminin-511 in the metastatic progression of breast cancer. Am. J. Pathol. *170*, 2135–2148.

Cho, N.K., Keyes, L., Johnson, E., Heller, J., Ryner, L., Karim, F., and Krasnow, M.A. (2002). Developmental control of blood cell migration by the Drosophila VEGF pathway. Cell *108*, 865–876.

Comber, K., Huelsmann, S., Evans, I., Sánchez-Sánchez, B.J., Chalmers, A., Reuter, R., Wood, W., and Martín-Bermudo, M.D. (2013). A dual role for the βPS integrin myospheroid in mediating Drosophila embryonic macrophage migration. J. Cell Sci. *126*, 3475–3484.

Denef, N., Chen, Y., Weeks, S.D., Barcelo, G., and Schüpbach, T. (2008). Crag regulates epithelial architecture and polarized deposition of basement membrane proteins in Drosophila. Dev. Cell *14*, 354–364.

Devergne, O., Sun, G.H., and Schüpbach, T. (2017). Stratum, a homolog of the human GEF Mss4, partnered with Rab8, controls the basal restriction of basement membrane proteins in epithelial cells. Cell Rep. *18*, 1831–1839.

Dunst, S., Kazimiers, T., von Zadow, F., Jambor, H., Sagner, A., Brankatschk, B., Mahmoud, A., Spannl, S., Tomancak, P., Eaton, S., and Brankatschk, M. (2015). Endogenously tagged rab proteins: a resource to study membrane trafficking in Drosophila. Dev. Cell *33*, 351–365.

Dutta, D., Bloor, J.W., Ruiz-Gomez, M., Viajay Raghavan, K., and Kiehart, D.P. (2002). Real-time imaging of morphogenetic movements in Drosophila using Gal4-UAS-driven expression of GFP fused to the actin-binding domain of Moesin. Genesis *34*, 416–151.

Evans, I.R., Hu, N., Skaer, H., and Wood, W. (2010). Interdependence of macrophage migration and ventral nerve cord development in Drosophila embryos. Development *137*, 1625–1633.

Fernández-Espartero, H., Ramel, D., Farago, M., Malartre, M., Luque, C.M., Limanovich, S., Katzav, S., Emery, G., and Marín-Bermudo, M.D. (2013). The GEF Vav regulates guided cell migration by coupling guidance receptor signaling to local Rac activation. J. Cell Science *126*, 2285–2293.

Frank, D.E., and Carter, W.G. (2004). Laminin 5 deposition regulates keratinocyte polarization and persistent migration. J. Cell Sci. *117*, 1351–1363.

Frydman, H.M., and Spradling, A.C. (2001). The receptor-like tyrosine phosphatase lar is required for epithelial planar polarity and for axis determination within Drosophila ovarian follicles. Development *128*, 3209–3220.

Haigo, S.L., and Bilder, D. (2011). Global tissue revolutions in a morphogenetic movement controlling elongation. Science *331*, 1071–1074.

Hamill, K.J., Kligys, K., Hopkinson, S.B., and Jones, J.C. (2009). Laminin deposition in the extracellular matrix: a complex picture emerges. J. Cell Sci. *122*, 4409–4417.

Hattula, K., Furuhjelm, J., Tikkanen, J., Tanhuanpää, K., Laakkonen, P., and Peränen, J. (2006). Characterization of the Rab8-specific membrane traffic route linked to protrusion formation. J. Cell Sci. *119*, 4866–4877.

Horwitz, R., and Webb, D. (2003). Cell migration. Curr. Biol. *13*, R756–R759.

Ishikawa, T., Wondimu, Z., Oikawa, Y., Gentilcore, G., Kiessling, R., Egyhazi Brage, S., Hansson, J., and Patarroyo, M. (2014). Laminins 411 and 421 differentially promote tumor cell migration via α6β1 integrin and MCAM (CD146). Matrix Biol. *38*, 69–83.

Jones, J.C., Lane, K., Hopkinson, S.B., Lecuona, E., Geiger, R.C., Dean, D.A., Correa-Meyer, E., Gonzales, M., Campbell, K., Sznajder, J.I., and Budinger, S. (2005). Laminin-6 assembles into multimolecular fibrillar complexes with perlecan and participates in mechanical-signal transduction via a dystroglycan-dependent, integrin-independent mechanism. J. Cell Sci. *118*, 2557–2566.

Kariya, Y., Sato, H., Katou, N., Kariya, Y., and Miyazaki, K. (2012). Polymerized laminin-332 matrix supports rapid and tight adhesion of keratinocytes, suppressing cell migration. PLoS ONE *7*, e35546.

Kawataki, T., Yamane, T., Naganuma, H., Rousselle, P., Andurén, I., Tryggvason, K., and Patarroyo, M. (2007). Laminin isoforms and their integrin receptors

in glioma cell migration and invasiveness: Evidence for a role of alpha5-laminin(s) and alpha3beta1 integrin. Exp. Cell Res. *313*, 3819–3831.

Kim, S.H., Turnbull, J., and Guimond, S. (2011). Extracellular matrix and cell signalling: the dynamic cooperation of integrin, proteoglycan and growth factor receptor. J. Endocrinol. *209*, 139–151.

Kusche-Gullberg, M., Garrison, K., MacKrell, A.J., Fessler, L.I., and Fessler, J.H. (1992). Laminin A chain: expression during *Drosophila* development and genomic sequence. EMBO J. *11*, 4519–4527.

Lebestky, T., Chang, T., Hartenstein, V., and Banerjee, U. (2000). Specification of Drosophila hematopoietic lineage by conserved transcription factors. Science *288*, 146–149.

Lee, H.-K., Wright, A.P., and Zinn, K. (2009). Live dissection of Drosophila embryos: streamlined methods for screening mutant collections by antibody staining. J. Vis. Exp. *34*, 1647.

Lerner, D.W., McCoy, D., Isabella, A.J., Mahowald, A.P., Gerlach, G.F., Chaudhry, T.A., and Horne-Badovinac, S. (2013). A Rab10-dependent mechanism for polarized basement membrane secretion during organ morphogenesis. Dev. Cell *24*, 159–168.

Mavor, L.M., Miao, H., Zuo, Z., Holly, R.M., Xie, Y., Loerke, D., and Blankenship, J.T. (2016). Rab8 directs furrow ingression and membrane addition during epithelial formation in Drosophila melanogaster. Development *143*, 892–903.

Mills, L., Tellez, C., Huang, S., Baker, C., McCarty, M., Green, L., Gudas, J.M., Feng, X., and Bar-Eli, M. (2002). Fully human antibodies to MCAM/MUC18 inhibit tumor growth and metastasis of human melanoma. Cancer Res. *62*, 5106–5114.

Mirre, C., Cecchini, J.-P., Le Parco, Y., and Knibiehler, B. (1988). De novo expression of a type IV collagen gene in Drosophila embryos is restricted to mesodermal derivatives and occurs at germ band shortening. Development *102*, 369–376.

Montell, D.J., and Goodman, C.S. (1989). Drosophila laminin: sequence of B2 subunit and expression of all three subunits during embryogenesis. J. Cell Biol. *109*, 2441–2453.

Oikawa, Y., Hansson, J., Sasaki, T., Rousselle, P., Domogatskaya, A., Rodin, S., Tryggvason, K., and Patarroyo, M. (2011). Melanoma cells produce multiple laminin isoforms and strongly migrate on α5 laminin(s) via several integrin receptors. Exp. Cell Res. *317*, 1119–1133.

Olofsson, B., and Page, D.T. (2005). Condensation of the central nervous system in embryonic Drosophila is inhibited by blocking hemocyte migration or neural activity. Dev. Biol. *279*, 233–243.

Palecek, S.P., Loftus, J.C., Ginsberg, M.H., Lauffenburger, D.A., and Horwitz, A.F. (1997). Integrin-ligand binding properties govern cell migration speed through cell-substratum adhesiveness. Nature *385*, 537–540.

Petrie, R.J., Doyle, A.D., and Yamada, K.M. (2009). Random versus directionally persistent cell migration. Nat. Rev. Mol. Cell Biol. *10*, 538–549.

Porther, N., and Barbieri, M.A. (2015). The role of endocytic Rab GTPases in regulation of growth factor signaling and the migration and invasion of tumor cells. Small GTPases *6*, 135–144.

Sarov, M., Barz, C., Jambor, H., Hein, M.Y., Schmied, C., Suchold, D., Stender, B., Janosch, S., K J, V.V., Krishnan, R.T., et al. (2016). A genome-wide resource for the analysis of protein localisation in Drosophila. eLife *5*, e12068.

Sehgal, B.U., DeBiase, P.J., Matzno, S., Chew, T.L., Claiborne, J.N., Hopkinson, S.B., Russell, A., Marinkovich, M.P., and Jones, J.C. (2006). Integrin beta4 regulates migratory behavior of keratinocytes by determining laminin-332 organization. J. Biol. Chem. *281*, 35487–35498.

Stramer, B., Moreira, S., Millard, T., Evans, I., Huang, C.Y., Sabet, O., Milner, M., Dunn, G., Martin, P., and Wood, W. (2010). Clasp-mediated microtubule bundling regulates persistent motility and contact repulsion in Drosophila macrophages in vivo. J. Cell Biol. *189*, 681–689.

Sung, B.H., and Weaver, A.M. (2011). Regulation of lysosomal secretion by cortactin drives fibronectin deposition and cell motility. Bioarchitecture *1*, 257–260.

Sung, B.H., Ketova, T., Hoshino, D., Zijlstra, A., and Weaver, A.M. (2015). Directional cell movement through tissues is controlled by exosome secretion. Nat. Commun. *6*, 7164.

Tepass, U., Fessler, L.I., Aziz, A., and Hartenstein, V. (1994). Embryonic origin of hemocytes and their relationship to cell death in Drosophila. Development *120*, 1829–1837.

Urbano, J.M., Torgler, C.N., Molnar, C., Tepass, U., López-Varea, A., Brown, N.H., de Celis, J.F., and Martín-Bermudo, M.D. (2009). Drosophila laminins act as key regulators of basement membrane assembly and morphogenesis. Development *136*, 4165–4176.

Wandinger-Ness, A., and Zerial, M. (2014). Rab proteins and the compartmentalization of the endosomal system. Cold Spring Harb. Perspect. Biol. *6*, a022616.

Wolfstetter, G., and Holz, A. (2012). The role of LamininB2 (LanB2) during mesoderm differentiation in Drosophila. Cell. Mol. Life Sci. *69*, 267–282.

Wood, W., Faria, C., and Jacinto, A. (2006). Distinct mechanisms regulate hemocyte chemotaxis during development and wound healing in Drosophila melanogaster. J. Cell Biol. *173*, 405–416.

Yasothornsrikul, S., Davis, W.J., Cramer, G., Kimberall, D.A., and Dearolf, C.R. (1997). identification and characterization of a second type IV collagen in Drosophila. Gene*198* (Viking), pp. 17–25.

Zhang, J., Schulze, K.L., Hiesinger, P.R., Suyama, K., Wang, S., Fish, M., Acar, M., Hoskins, R.A., Bellen, H.J., and Scott, M.P. (2007). Thirty-one flavors of Drosophila rab proteins. Genetics *176*, 1307–1322.

Cell Reports, Volume *21*

Supplemental Information

Drosophila Embryonic Hemocytes Produce Laminins to Strengthen Migratory Response

Besaiz J. Sánchez-Sánchez, José M. Urbano, Kate Comber, Anca Dragu, Will Wood, Brian Stramer, and María D. Martín-Bermudo

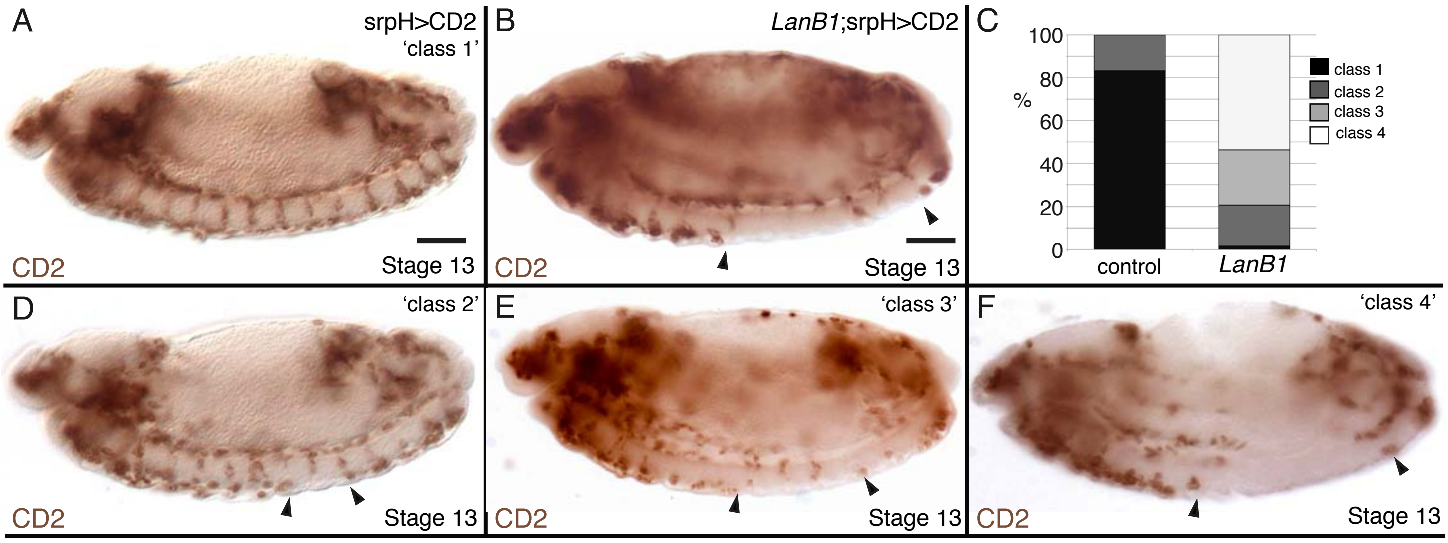

Supplemental Figure 1 (related to Figure 1): Hemocyte migration is defective in the absence of laminins.

(A, B, D-F) Lateral view of fixed and stained stage 13 embryos. Hemocytes are visualized by expression of the heterologous cell membrane marker CD2 driven by the srpH-Gal4 driver and detected with an anti-CD2 antibody. (A) Control embryo. (B) LanB1 embryo. (C) Quantification of hemocyte migration phenotype over the VNC. This was done accordingly to (Comber et al., 2013). To summarize, we used four distinct phenotypic classes, according to the number of neuromites of the VNC devoid of hemocytes: 'class 1': 0 neuromites, 'class 2': 1-2 neuromites, 'class 3': 3-4 neuromites and 'class 4': ≥ 5 neuromites. (D-F) Grading of LanB1 embryos into 'classes' based on the migration defects along the VNC.

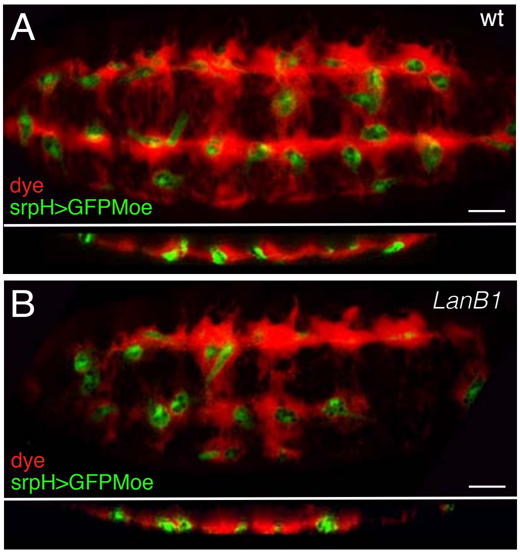

Supplemental Figure 2 (related to Figuress 1 and 2): Hemocyte migration along the VNC: environmental requirements for laminins.

(A, B) Orthogonal projections of ventrally orientated stage 15 control and LanB1 embryos expressing GFP in hemocytes (green) injected with dextran dye (red) to reveal spatial constraints surrounding the hemocytes. While in control embryos (A), the dye permeates along the length of the embryo, indicating proper VNC-epithelial separation, in LanB1 embryos (B), spreading of the dye becomes restricted, demonstrating incomplete VNC-epithelial separation. Scale bar represents 50µm.

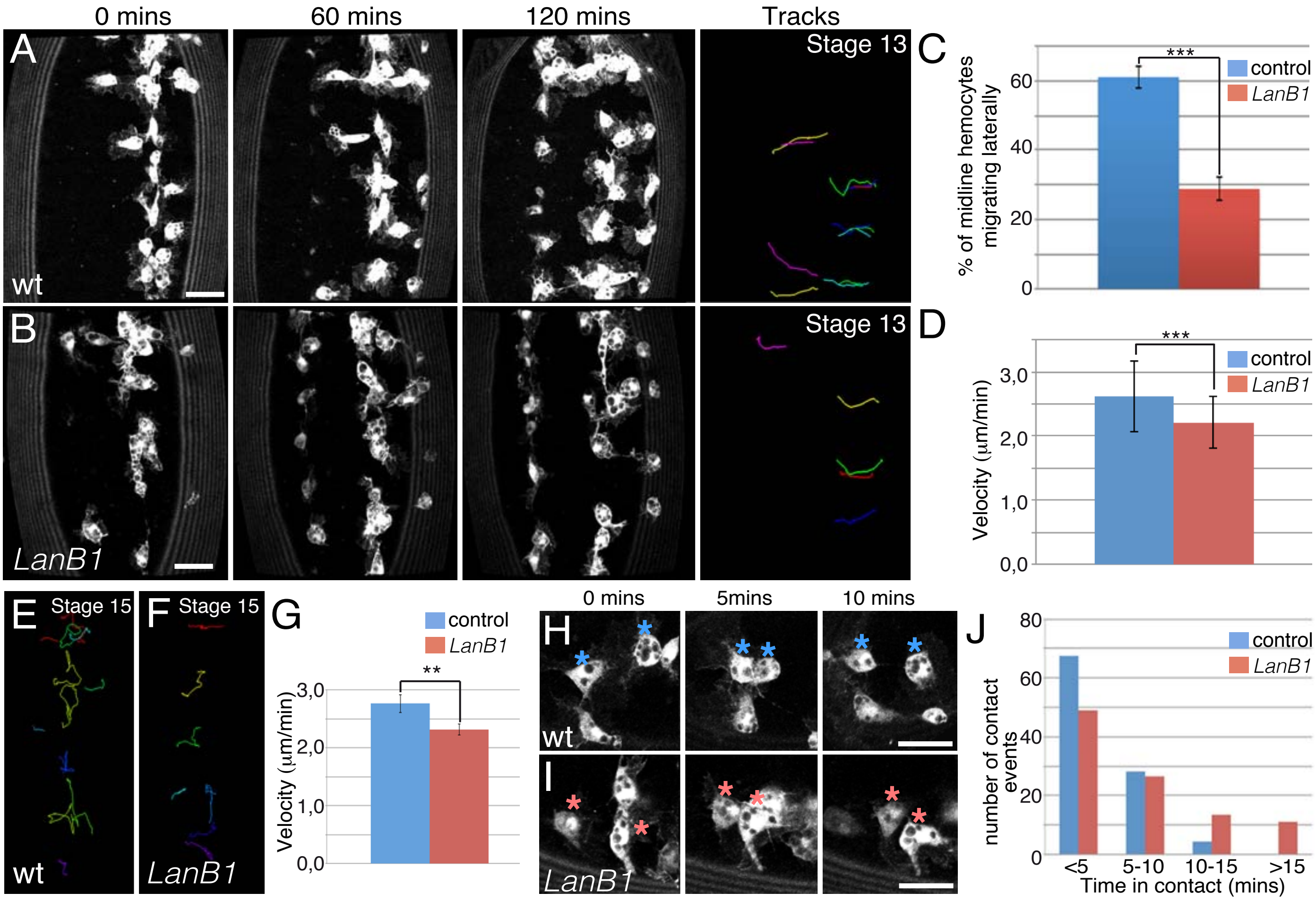

Supplemental Figure 3 (related to Figures 1 and 2): Laminins regulate hemocyte lateral and random migrations

(A and B) Still images taken from live imaging of hemocytes expressing GFPMoe undergoing lateral migration at stage 13/14. (C) Tracking hemocytes undergoing lateral migration reveals a decrease in the percentage of hemocytes migrating laterally in LanB1 embryos (average decrease from 62.2% and 29.4% $p<0.001$, n=5 embryos per genotype) and (D) a significant decrease in the speed of the hemocytes that did (average velocity of hemocytes from control and LanB1 embryos were 2.62 ± 0.56 µm/min and 2.21 ± 0.40 µm/min respectively, $p<0.001$). (E, F) Tracking of GFP expressing hemocytes undergoing random migration at stage 15 reveals a slight but statistically significant reduction in random migration velocity of hemocytes from LanB1 embryos compared to controls (G). Hemocytes from control and LanB1 embryos migrated at 2.76 ± 0.15µm/min and 2.31 ± 0.09µm/min respectively, $p<0.01$). (H, I) Stills taken from live imaging of GFP expressing hemocytes in control and LanB1 mbryos undergoing cell-cell contact repulsion at stage 15. Hemocytes remain in contact for longer periods in LanB1 embryos (I, pink asterisks) than in control embryos (H, blue asterisks). (J) Quantification of the time the lamellipodia of two hemocytes remain in contact indicates an increase in LanB1 embryos (average time in contact for hemocytes from control and LanB1 embryos was 4.9 and 7.6 mins respectively, $p<0.001$, n=91 (control) and n=100 (LanB1) contact events). Scale bar represents 20 µm.

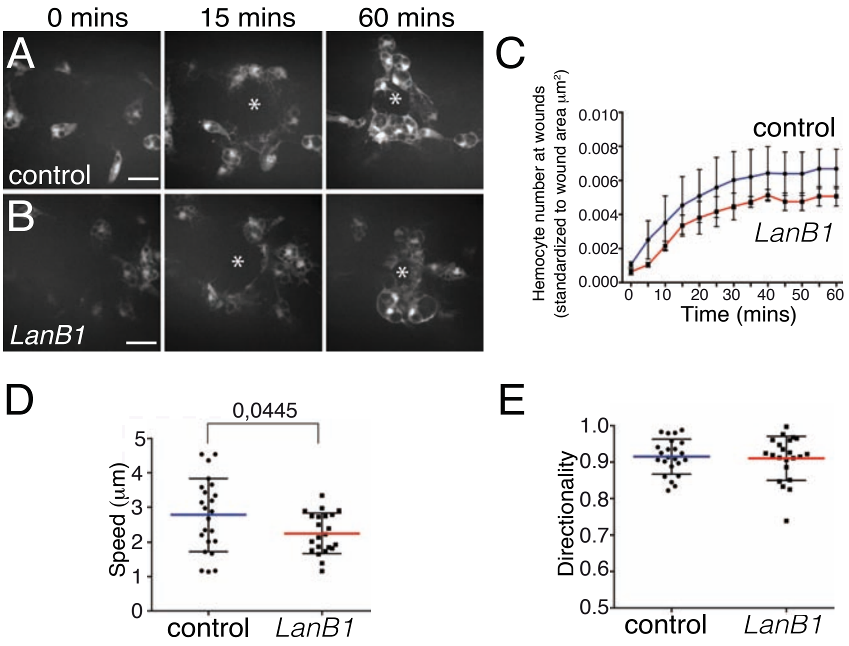

Supplemental Figure 4 (related to Figure 1): Laminins are required for inflammatory migration

(A, B) Stills taken from movies of hemocytes in control (A) and LanB1 (B) embryos migrating to an epithelial wound (asterisk). Scale bar represents. (C) Monitoring the number of hemocytes at the wound every 5 minutes post wounding over a 60 minute time period reveals a small but significant reduction in the number of hemocytes present at early time points following wounding in LanB1 embryos compared to control ($p<0.05$ at 10 and 15 mins post wounding). (D) In addition, tracking reveals that hemocytes from LanB1 embryos migrate towards a wound at lower speed than those from controls (2.3 ± 0.6μm/min and 3.0 ± 0.3μm/min respectively, $p<0.05$, n=23 (control) and n=22 (LanB1) hemocytes). Scale bar represents 25μm.

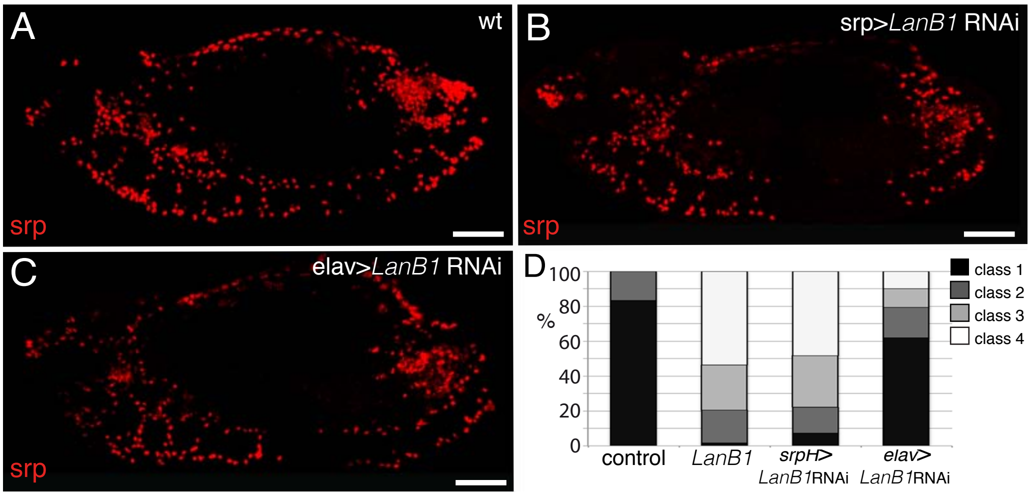

Supplemental Figure 5 (related to Figures 1 and 2): Hemocyte and neuronal specific requirements for laminins

(A-C) Lateral view of fixed stage 13 embryos stained with an anti-Srp antibody (red). (A) Wild type embryo and LanB1 RNAi specific expression in either hemocytes (B) or the VNC (C). Down-regulation of LanB1 levels specifically in hemocytes (B), but not in neuronal and glial cells (C), results in migration defects similar to those observed in LanB1 embryos. (D) Quantification of the hemocyte migration phenotype in embryos of the indicated genotypes. Scale bar represents 50µm.

Supplemental References

Comber, K., S. Huelsmann, I. Evans, B.J. Sanchez-Sanchez, A. Chalmers, R. Reuter, W. Wood, and M.D. Martin-Bermudo. 2013. A dual role for the betaPS integrin myospheroid in mediating Drosophila embryonic macrophage migration. *J Cell Sci*. 126:3475-3484.
